# Real-Time EEG Decoding of Motor Imagery via Nonlinear Dimensionality Reduction (Manifold Learning) and Shallow Classifiers

**DOI:** 10.3390/bios15100692

**Published:** 2025-10-13

**Authors:** Hezzal Kucukselbes, Ebru Sayilgan

**Affiliations:** 1 Department of Electrical and Electronics Engineering, Izmir University of Economics, Izmir 35330, Turkey; hezzalkucuk@gmail.com; 2 Department of Mechatronics Engineering, Izmir University of Economics, Izmir 35330, Turkey

**Keywords:** SCI, EEG, BCI, rehabilitation systems, manifold learning

## Abstract

This study introduces a real-time processing framework for decoding motor imagery EEG signals by integrating manifold learning techniques with shallow classifiers. EEG recordings were obtained from six healthy participants performing five distinct wrist and hand motor imagery tasks. To address the challenges of high dimensionality and inherent nonlinearity in EEG data, five nonlinear dimensionality reduction methods, t-SNE, ISOMAP, LLE, Spectral Embedding, and MDS, were comparatively evaluated. Each method was combined with three shallow classifiers (*k*-NN, Naive Bayes, and SVM) to investigate performance across binary, ternary, and five-class classification settings. Among all tested configurations, the t-SNE + *k*-NN pairing achieved the highest accuracies, reaching 99.7% (two-class), 99.3% (three-class), and 89.0% (five-class). ISOMAP and MDS also delivered competitive results, particularly in multi-class scenarios. The presented approach builds upon our previous work involving EEG datasets from individuals with spinal cord injury (SCI), where the same manifold techniques were examined extensively. Comparative findings between healthy and SCI groups reveal consistent advantages of t-SNE and ISOMAP in preserving class separability, despite higher overall accuracies in healthy subjects due to improved signal quality. The proposed pipeline demonstrates low-latency performance, completing signal processing and classification in approximately 150 ms per trial, thereby meeting real-time requirements for responsive BCI applications. These results highlight the potential of nonlinear dimensionality reduction to enhance real-time EEG decoding, offering a low-complexity yet high-accuracy solution applicable to both healthy users and neurologically impaired individuals in neurorehabilitation and assistive technology contexts.

## 1. Introduction

Every year, spinal cord injuries (SCIs) affect approximately 250,000 to 500,000 individuals globally, with an estimated two to three million people living with SCI-related disabilities [1]. SCI arises from damage to the spinal cord or surrounding structures, disrupting communication between the brain and body [2]. Causes include traumatic incidents such as vehicular accidents, falls, and sports injuries, as well as non-traumatic factors. Clinical manifestations vary depending on the injury’s severity and location, commonly resulting in sensory and motor impairments, muscular weakness, and complications in physiological functions [3]. While complete injuries typically lead to permanent deficits, partial injuries may permit some functional recovery.

Technological advancements have significantly improved rehabilitation approaches and patient quality of life. Among these, brain-computer interfaces (BCIs) that leverage electroencephalography (EEG) have emerged as promising tools. EEG-based BCIs enable direct communication between the brain and external devices, offering a non-invasive, portable, and cost-effective solution for individuals with limited motor control [4,5]. EEG signals, which capture oscillatory neural activity, are acquired via electrodes placed on the scalp. These signals can be decoded in real time using machine learning algorithms to infer user intentions [6].

Despite their potential, EEG signals present analytical challenges due to their high dimensionality, low signal-to-noise ratio, and variability across sessions and subjects [7]. Effective dimensionality reduction is thus essential for improving signal decoding accuracy and computational efficiency. Traditional techniques like Principal Component Analysis (PCA) have been widely used, but recent research focuses on nonlinear methods better suited to the intrinsic geometry of EEG data [8].

Manifold learning is a powerful class of nonlinear dimensionality reduction methods that seeks low-dimensional representations while preserving local or global data structures [9,10,11]. These methods are particularly advantageous in processing EEG data due to their ability to retain discriminative features critical for classification [12]. Prominent manifold learning algorithms include ISOMAP [11], Locally Linear Embedding (LLE) [10], t-Distributed Stochastic Neighbor Embedding (t-SNE) [13], Spectral Embedding [14], and Multidimensional Scaling (MDS) [15].

In recent years, the integration of manifold learning techniques with shallow classifiers such as k-nearest neighbors (*k*-NN), Support Vector Machines (SVMs), and Naive Bayes has shown promise in decoding motor imagery (MI) tasks from EEG [16,17]. These combinations enable efficient real-time EEG decoding with reduced computational burden. Moreover, comparative studies suggest that manifold learning can improve classification accuracy in EEG-based BCIs, particularly for applications in neurorehabilitation and assistive technology [18].

This study aims to explore the effectiveness of manifold learning techniques paired with shallow classifiers for classifying EEG data collected from six healthy participants performing five wrist and hand motor imagery tasks. The performance of various dimensionality reduction-classifier pairs is evaluated across binary, ternary, and five-class scenarios to identify robust, low-complexity pipelines suitable for real-time BCI applications.

### 1.1. State of the Art

Recent studies have highlighted the potential of manifold learning and advanced feature extraction techniques in enhancing the classification performance of EEG-based BCIs, particularly in motor imagery tasks.

Li et al. [19] introduced an adaptive feature extraction framework combining wavelet packet decomposition (WPD) and semidefinite embedding ISOMAP (SE-ISOMAP). This approach utilized subject-specific optimal wavelet packets to extract time-frequency and manifold features, achieving 100% accuracy in binary classification tasks and significantly outperforming conventional dimensionality reduction methods.

Yamamoto et al. [20] proposed a novel method called Riemann Spectral Clustering (RiSC), which maps EEG covariance matrices as graphs on the Riemannian manifold using a geodesic-based similarity measure. They further extended this framework with odenRiSC for outlier detection and mcRiSC for multimodal classification, where mcRiSC reached 73.1% accuracy and outperformed standard single-modal classifiers in heterogeneous datasets.

Krivov and Belyaev [21] incorporated Riemannian geometry and Isomap to reveal the manifold structure of EEG covariance matrices in a low-dimensional space. Their method, evaluated with Linear Discriminant Analysis (LDA), reported classification accuracies of 0.58 (CSP), 0.61 (PGA), and 0.58 (Isomap) in a four-class task, underlining the potential of manifold methods in representing EEG data structures.

Tyagi and Nehra [22] compared LDA, PCA, FA, MDS, and ISOMAP for motor imagery feature extraction using BCI Competition IV datasets. A feedforward artificial neural network (ANN) trained with the Levenberg-Marquardt algorithm yielded the lowest mean square error (MSE) with LDA (0.1143), followed by ISOMAP (0.2156), while other linear methods showed relatively higher errors.

Xu et al. [23] designed an EEG-based attention classification method utilizing Riemannian manifold representation of symmetric positive definite (SPD) matrices. By integrating amplitude and phase information using a filter bank and applying SVM, their approach reached a classification accuracy of 88.06% in a binary scenario without requiring spatial filters.

Lee et al. [24] assessed the efficacy of PCA, LLE, and ISOMAP in binary EEG classification using LDA. The classification errors were reported as 28.4% for PCA, 25.8% for LLE, and 27.7% for ISOMAP, suggesting LLE’s slight edge in capturing intrinsic EEG data structures.

Li, Luo, and Yang [25] further evaluated the performance of linear and nonlinear dimensionality reduction techniques in motor imagery EEG classification. Nonlinear methods such as LLE (91.4%) and parametric t-SNE (94.1%) outperformed PCA (70.7%) and MDS (75.7%), demonstrating the importance of preserving local neighborhood structures for robust feature representation.

Sayılgan [26] investigated EEG-based classification of imagined hand movements in spinal cord injury patients using Independent Component Analysis (ICA) for feature extraction and machine learning classifiers including SVM, *k*-NN, AdaBoost, and Decision Trees. The highest accuracy was achieved with SVM (90.24%), while *k*-NN demonstrated the lowest processing time, with the lateral grasp showing the highest classification accuracy among motor tasks.

These studies collectively underline the critical role of dimensionality reduction, particularly manifold learning, in effectively decoding motor intentions from EEG data, thereby improving the performance and applicability of BCI systems in neurorehabilitation contexts.

### 1.2. Contributions

EEG recordings, collected from multiple scalp locations, are inherently high-dimensional, often containing redundant information and being susceptible to various noise sources and artifacts. Such properties can hinder the accuracy and robustness of motor intention decoding in brain-computer interface (BCI) systems. While conventional linear dimensionality reduction approaches, such as Principal Component Analysis (PCA) and Linear Discriminant Analysis (LDA), are widely used, they often fail to capture the complex, nonlinear temporal-spatial relationships embedded in EEG activity patterns. In contrast, manifold learning techniques offer a promising alternative by projecting data into a lower-dimensional space while preserving its intrinsic local geometry.

In this work, we present a comprehensive and adaptable manifold learning-based processing framework for EEG analysis, designed to support the development of rehabilitation-oriented BCIs. The proposed pipeline integrates multiple nonlinear dimensionality reduction algorithms with shallow classifiers to alleviate overfitting, enhance inter-class separability, and improve overall decoding performance.

The main contributions of this study can be outlined as follows:

- Introduction of a unified manifold learning framework for the classification of motor imagery EEG signals into binary (two-class), ternary (three-class), and multi-class (five-class) categories, using real-time data acquired from healthy participants.
- Systematic comparison of five widely recognized manifold learning algorithms: Spectral Embedding, Locally Linear Embedding (LLE), Multidimensional Scaling (MDS), Isometric Mapping (ISOMAP), and t-distributed Stochastic Neighbor Embedding (t-SNE) for their effectiveness as feature transformation tools in motor intent recognition.
- Alignment of the proposed methodology with practical rehabilitation needs, specifically for integration into a cost-efficient, two-degree-of-freedom robotic platform employing a straightforward control strategy.
- Emphasis on building a sustainable machine learning model capable of accurately detecting motor intentions in healthy users while ensuring high classification performance, thereby enabling scalability to clinical scenarios.
- Addressing a notable gap in the literature by exploring high-accuracy three-class and five-class EEG-based BCI paradigms for spinal cord injury (SCI) rehabilitation, and benchmarking binary classification results against state-of-the-art systems.
- Analysis of task combination compatibility across different classification schemes, with performance metrics aggregated over all participants to support model generalizability.
- Comprehensive evaluation using multiple performance indicators—accuracy, precision, recall, and F1-score-to assess the robustness of each manifold-classifier pairing under varying task complexities.

## 2. Materials and Methods

### 2.1. EEG Experimental Procedure

In this study, real-time EEG signals were recorded from six healthy participants (see Table 1) using the OpenBCI “All-in-One EEG Electrode Cap Bundle”. The system includes the Cyton + Daisy biosensor boards, a 19-channel electrode cap with Ag/AgCl-coated electrodes, a USB wireless dongle, a 6 V AA battery pack, a Head Pin Touch Adapter (HPTA), electrode gel, and associated accessories. The Cyton board offers 8 channels of EEG data acquisition, which is extended to 16 channels using the Daisy module, operating at a sampling rate of 250 Hz per channel [27].

**Figure 1:**
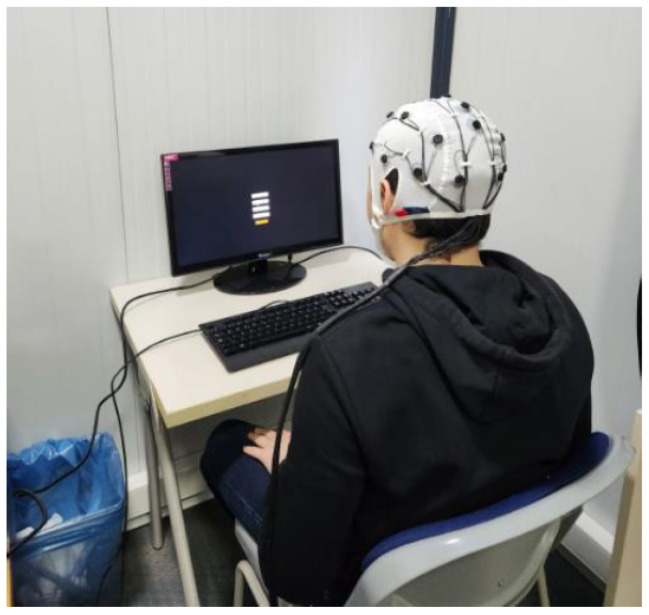
Motor imagery EEG experiment setup showing a participant with an OpenBCI EEG cap interacting with the Unity-based stimulus interface.

Before the experiment, the electrode cap was positioned on the participant’s head according to the 10–20 international placement system. Electrode gel was applied to each electrode location using a syringe to ensure optimal conductivity. The cap’s electrodes were then connected to the input pins of the Cyton and Daisy boards, which were wirelessly linked to the recording computer via the OpenBCI USB dongle. Figure 1 illustrates the setup procedure.

**Figure 2:**
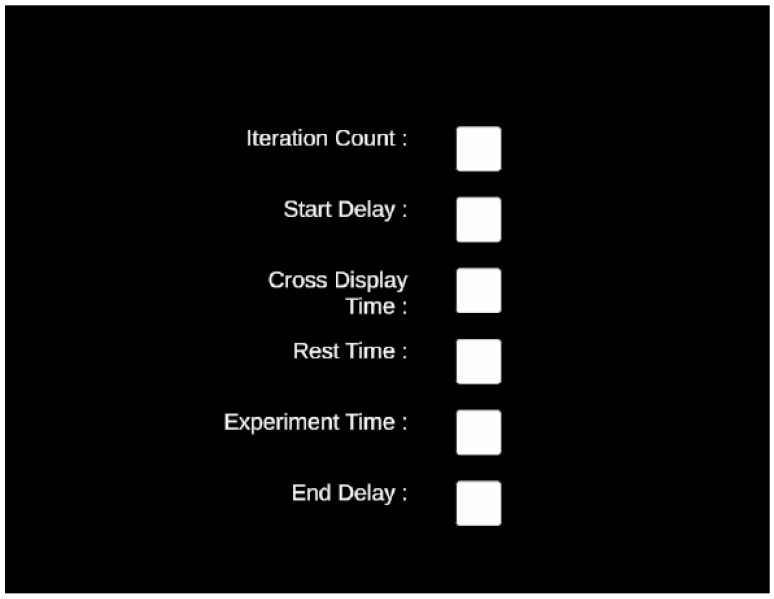
Visual components of the EEG experimental protocol. The parameter configuration interface is used before each session.

**Figure 3:**
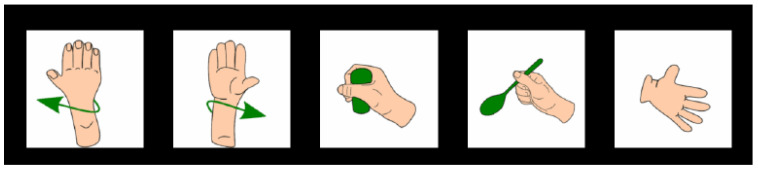
Visual stimuli were shown to participants to prompt imagined motor execution [28].

The experimental paradigm was implemented using a custom interface developed in Unity (Figure 2). After the participant’s information was entered and a specific hand movement class was selected (Figure 3). The particular hand movement classes were inspired by the work of Ofner et al. [28], who demonstrated their effectiveness in studies involving patients with spinal cord injury (SCI). The experiment began with a predefined sequence of visual stimuli. The protocol consisted of five repetitions per class, with a 3 s start delay, a 5 s stimulus duration (fixation cross), followed by a 3 s rest period. Each trial concluded with a 3 s post-stimulus delay.

**Figure 4:**
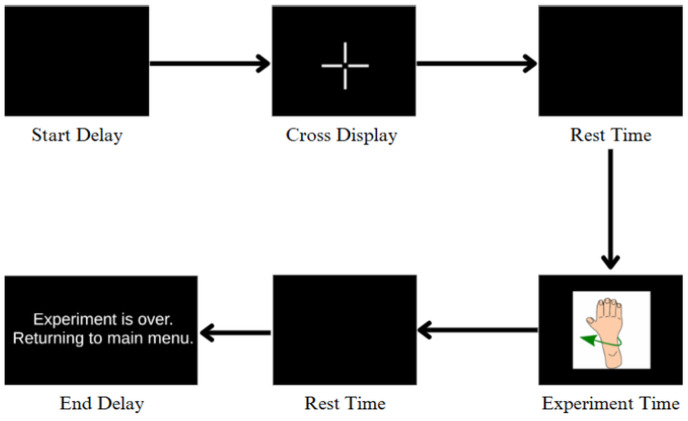
Flow diagram of the EEG experimental protocol. Each trial begins with a start delay followed by a fixation cross display to direct participant focus. A rest period is then introduced before the motor imagery stimulus (i.e., hand movement) is shown. After the participant imagines the movement, another rest period is initiated. Finally, an end delay marks the conclusion of the iteration with an exit message.

During each trial, participants were instructed to perform motor imagery of the displayed hand movement (e.g., left hand open, right hand grasp) while minimizing physical motion. The experimental flow involved an initial blank screen, followed by the appearance of a fixation cross, a rest interval, and finally the visual cue corresponding to the intended movement (Figure 4). Upon completion of all repetitions, a message indicating the end of the experiment was displayed.

The EEG recording process was initiated simultaneously with the start of the experiment via the OpenBCI GUI, allowing synchronized signal acquisition and stimulus presentation. The paradigm is aligned with standard motor imagery-based BCI protocols, where subjects are encouraged to mentally rehearse the movement in the absence of actual execution, which has been shown to activate relevant cortical motor areas [29,30].

### 2.2. EEG Dataset Description and Preprocessing

EEG signals were acquired from six healthy volunteers performing five motor imagery tasks. EEG signals were acquired from six healthy volunteers performing five motor imagery tasks. The recordings were collected using a 32-channel EEG acquisition system (BrainVision Recorder, Brain Products GmbH, Gilching, Germany, v1.22) at a sampling rate of 512 Hz. Data preprocessing and feature extraction were conducted using MATLAB R2024a (MathWorks, Natick, MA, USA) [31], and machine learning pipelines were implemented on Orange Data Mining v3.36 [32]. All software versions and references are provided to ensure reproducibility.

**Figure 5:**
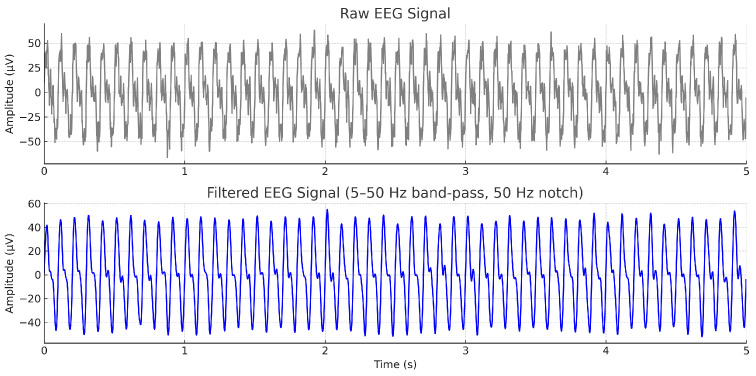
Example EEG signal from a representative channel before (**top**) and after (**bottom**) preprocessing. A 5–50 Hz band-pass filter and a 50 Hz notch filter were applied to preserve motor-related rhythms (mu: 8–13 Hz, beta: 13–30 Hz, low gamma: 30–50 Hz) and remove power-line noise.

The raw EEG signals acquired through the OpenBCI GUI platform were initially subjected to preprocessing operations within the same platform. A band-pass filter was applied to retain frequencies within a specified range, allowing only signals between 5.0 Hz and 50.0 Hz to pass, shown in Figure 5. This frequency window effectively preserves relevant neural oscillations typically associated with motor tasks, including the mu (8–13 Hz), beta (13–30 Hz), and low gamma (30–50 Hz) bands, while attenuating lower-frequency drifts and high-frequency noise.

Subsequently, a notch filter was employed to suppress power line interference typically observed around 50 Hz to 60 Hz, thereby improving signal fidelity. A fourth-order Butterworth filter was selected as the filter type due to its advantageous characteristics, including minimal phase distortion, smooth frequency response, and flat passband behavior. The choice of a fourth-order filter ensures adequate sharpness in the transition band while maintaining the integrity of the EEG signal structure, which is crucial for subsequent feature extraction and classification tasks [7,28].

The active task intervals were identified as the 9–13th, 25–29th, 41–45th, 57–61st, and 73–77th seconds of each trial. Each signal was then normalized using z-score normalization, a widely adopted technique in EEG signal preprocessing [8]. The formula is defined as follows:

(1) $$Z=\frac{X-\mu }{\sigma }$$

where *X* denotes an individual EEG data point, μ represents the mean of the EEG channel, and σ is the standard deviation of the same channel.

**Figure 6:**
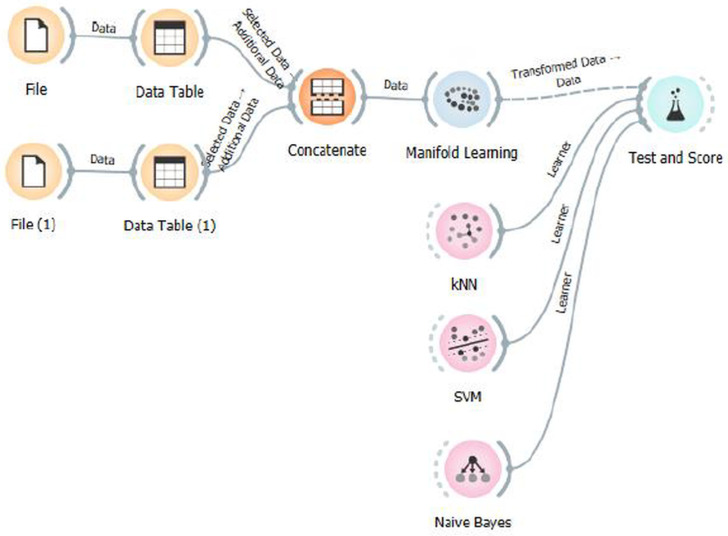
Workflow of the classification pipeline implemented in Orange Data Mining software. Raw EEG datasets are imported and preprocessed through individual Data Table modules, then concatenated into a unified dataset. Manifold learning methods are applied for dimensionality reduction, followed by classification using k-nearest neighbors (*k*-NN), Support Vector Machine (SVM), and Naive Bayes classifiers. The final evaluation is performed using the Test and Score module.

After normalization, all combinations of tasks related to motor imagery in grades 2, 3, and 5 were constructed, and the results were evaluated using a machine learning pipeline (Figure 6) implemented in the Orange Data Mining platform [32].

### 2.3. Manifold Learning Methods

#### 2.3.1. Multi-Dimensional Scaling (MDS)

Multi-Dimensional Scaling (MDS) is a non-linear, unsupervised dimensionality reduction technique that represents the pairwise dissimilarities between high-dimensional data points in a lower-dimensional space while preserving their relative distances [33]. MDS is commonly employed in exploratory and multivariate data analysis and has gained considerable attention in recent years [34]. There are three main variants of MDS-classical, metric, and non-metric each suited to different types of dissimilarity data and analysis objectives [35].

In classical MDS, the pairwise distances between samples in the input data matrix *X* are computed using the Euclidean distance metric, which is most appropriate for quantitative data. The Euclidean distance between two data points $x_{i}$ and $x_{j}$ in a high-dimensional space is calculated as

(2) $$d\left(x_{i},x_{j}\right)=\sqrt{\sum_{k=1}^{n}{\left(x_{ik}-x_{jk}\right)}^{2}}$$

The classical MDS algorithm consists of two primary matrices: the squared distance matrix $D^{2}$ and the centering matrix *H*. The matrix $D^{2}$ contains squared Euclidean distances, while *H* is defined as

(3) $$H=I-\frac{1}{n}11^{⊤}$$

where *I* is the identity matrix of size n×n, and 1 is a column vector of ones of length *n*.

(4) $$B=-\frac{1}{2}HD^{2}H$$

The final step involves the eigenvalue decomposition of the matrix *B*, yielding a set of eigenvalues and eigenvectors. The coordinates in the reduced-dimensional space are then obtained by

(5) $$Y=U\Lambda^{1/2}$$

where *U* is the matrix of eigenvectors and Λ is the diagonal matrix of eigenvalues. This transformation allows the high-dimensional data to be embedded into a low-dimensional space that preserves the original pairwise distances as closely as possible [36].

#### 2.3.2. Isometric Feature Mapping (ISOMAP)

Isometric Feature Mapping (ISOMAP) is a non-linear dimensionality reduction technique that extends classical Multi-Dimensional Scaling (MDS) by incorporating geodesic distances rather than Euclidean distances. In contrast to linear methods, ISOMAP is designed to reveal the intrinsic geometry of high-dimensional data lying on a non-linear manifold by approximating the true geodesic distances between all pairs of points [11,37,38].

The algorithm operates through three main steps:

Step 1: Construction of the Neighborhood Graph

A neighborhood graph is constructed to represent local relationships among data points. This can be achieved using either the ϵ-neighborhood criterion, where each point is connected to all others within a fixed radius ϵ, or the *k*-nearest neighbors (*k*-NN) approach, where each point is connected to its *k* closest neighbors. The resulting graph is weighted, with edges reflecting Euclidean distances between neighboring points, denoted as $d_{x}\left(i,j\right)$. This graph approximates the local structure of the underlying manifold [11].

Step 2: Estimation of Geodesic Distances

In manifold learning, the shortest path between two points is measured along the curved surface of the manifold, referred to as the geodesic distance. ISOMAP approximates these distances by computing the shortest path through the neighborhood graph using algorithms such as Dijkstra’s or Floyd-Warshall. This results in a geodesic distance matrix $D_{G}$, which approximates the pairwise intrinsic distances between all data points [39].

Step 3: Application of Classical MDS

Once the geodesic distance matrix $D_{G}$ is computed, classical MDS is applied to this matrix to generate a low-dimensional embedding. The goal is to find a mapping $Y\in R^{m}$ that best preserves the pairwise geodesic distances in the reduced space. This step involves double-centering of the matrix and eigenvalue decomposition, similar to classical MDS, but using $D_{G}$ instead of Euclidean distances [38,40].

ISOMAP thus enables the unfolding of complex manifolds by preserving global geometric structures and providing a meaningful low-dimensional representation suitable for classification, visualization, or further analysis.

#### 2.3.3. Local Linear Embedding (LLE)

The Locally Linear Embedding (LLE) is recognized as an unsupervised learning algorithm through which a low-dimensional embedding of high-dimensional inputs is computed, with neighborhood relationships being preserved [41].

In contrast to clustering techniques that are employed to reduce the dimensionality of local data, LLE effectively transforms its inputs into a singular global coordinate system that possesses a lower dimensionality. Furthermore, the optimization processes of LLE are not subject to local minimum. LLE’s employment of local symmetries inherent in linear reconstructions facilitates the acquisition of knowledge concerning the overall structure of nonlinear manifolds. This capability is particularly pronounced in scenarios where these manifolds are derived from facial images or textual documents [10].

LLE endeavors to preserve the local affine structure. Under the assumption that each data point lies within a manifold’s locally linear patch, LLE characterizes the local geometry by representing each data point as an approximate affine combination of its neighboring points. After this process, LLE accomplishes a reduction in dimension through the construction of a scatter of points in low dimensions, ensuring the optimal maintenance of the affine combination coefficients obtained from the high-dimensional data space [42].

It is required to find the *k*-NN for each data point $x_{i}$ in the data set. This particular process is founded on the assumption that a small linear segment on a larger manifold is formed collectively by these data points. In the context of these local segments, it is possible to establish linear coefficients that facilitate the reconstruction of each observation based on its neighboring observations. The accuracy of the reconstructed data can be determined by calculating the total squared dissimilarity between each data point and the reconstruction based on that point. In addition, the weight matrix *W* can be determined by minimizing the sequence error.

(6) $$E\left(W\right)=\sum_{i=1}^{n}{\left|X_{i}-\sum_{j=1}^{n}W_{ij}X_{j}\right|}^{2}$$

The process involves the computation of the squared distances between all data points and their respective reconstructions. The contribution of each data point to the reconstruction of the corresponding point is represented by the assigned weights. The calculation of the weights $W_{ij}$ is done by minimizing the cost function, subject to two restrictions. The first constraint specifies that each data point $X_{i}$ is reconstructed exclusively from its neighbors, thereby forcing

(7) $$W_{ij}=0$$

if $X_{j}$ does not belong to this set. The index *j* in the equation indicates the data points that are located within the k-nearest neighbors of the data point $X_{i}$, where the optimal weights in the error function are obtained using the least squares method under the constraint that the sum of the rows of the weight matrix equals one:

(8) $$\sum_{i=1}^{n}W_{ij}=1$$

In the final phase of the algorithm, each high-dimensional observation, denoted $X_{i}$, is mapped to a low-dimensional vector denoted $Y_{i}$ which denotes global internal coordinates on the manifold. This process is achieved by selecting d-dimensional coordinates, denoted as $Y_{i}$, to minimize the embedding cost function, thereby ensuring the optimal representation of the data.

(9) $$\Phi \left(Y\right)=\sum_{i=1}^{n}{\left|Y_{i}-\sum_{j=1}^{n}W_{ij}Y_{j}\right|}^{2}$$

The sparse eigenvalue-eigenvector approach is a viable method to solve the minimization problem. The procedure for obtaining a symmetric and semi-positive N*x*.

N-dimensional sparse matrix for eigenvalue decomposition is described in equation form.

(10) $$M=\left(I-W\right){\left(I-W\right)}^{T}$$

Independent coordinates centered at the origin are provided by the eigenvectors associated with the smallest non-zero eigenvalue *M* of the *d*-matrix.

The LLE can be summarizen as follows: (1) The determination of *k* for the neighborhood, as well as the number of dimensions *d* in the reduced coordinate system, is required. Then the neighbors of each data point $X_{i}$ are calculated based on the selected *k*. (2) The weights $W_{ij}$, which represent the contribution of each data point $X_{i}$ to its nearest neighbors, are calculated by reconstructing $X_{i}$ as a linear combination of its neighbors. These weights are calculated by minimizing the cost function through constrained linear optimization to ensure the most accurate representation of $X_{i}$ in its local neighborhood. (3) The points $Y_{i}$ are constructed in reduced d-dimensional space to ensure that the weights $W_{ij}$ remain consistent. This is achieved by computing the vectors $Y_{i}$ that can best be reconstructed by $W_{ij}$, minimizing the quadratic form in the equation using the smallest non-zero eigenvectors [10,36,43].

#### 2.3.4. t-Distributed Stochastic Neighbor Embedding (t-SNE)

t-Distributed Stochastic Neighbor Embedding (t-SNE) is a nonlinear, unsupervised dimensionality reduction technique introduced by Laurens van der Maaten and Geoffrey Hinton in 2008. It has been widely employed for the visualization and exploration of high-dimensional data, particularly due to its ability to preserve local neighborhood structures when projecting data into a lower-dimensional space.

t-SNE minimizes the Kullback-Leibler (KL) divergence between two probability distributions: one that measures pairwise similarities of data points in the high-dimensional space and another that measures pairwise similarities in the low-dimensional embedding. This cost function is non-convex, and therefore, different initializations may lead to different solutions. Nonetheless, t-SNE is particularly effective at revealing clusters and complex data structures at multiple scales [44].

The algorithm operates in three main steps:

##### Step 1: Compute High-Dimensional Similarities

A Gaussian distribution centered at each data point $x_{i}$ is used to model pairwise similarities in the high-dimensional space. The conditional probability $p_{j|i}$ indicates the likelihood that point $x_{j}$ would be a neighbor of $x_{i}$:

(11) $$p_{j|i}=\frac{\exp\left(-\frac{{∥x_{i}-x_{j}∥}^{2}}{2\sigma_{i}^{2}}\right)}{\sum_{\begin{array}{c} k=1\\ k\neq i \end{array}}^{n}\exp\left(-\frac{{∥x_{i}-x_{k}∥}^{2}}{2\sigma_{i}^{2}}\right)}$$

To obtain a symmetric joint probability, the following is computed:

(12) $$p_{ij}=\frac{p_{j|i}+p_{i|j}}{2N}$$

where *N* is the total number of data points.

##### Step 2: Compute Low-Dimensional Similarities

In the low-dimensional space, the similarity between points $y_{i}$ and $y_{j}$ is modeled using a heavy-tailed Student’s t-distribution with one degree of freedom:

(13) $$q_{ij}=\frac{{\left(1+{∥y_{i}-y_{j}∥}^{2}\right)}^{-1}}{\sum_{\begin{array}{c} i=1,\\ k\neq i \end{array}}^{n}{\left(1+{∥y_{i}-y_{k}∥}^{2}\right)}^{-1}}$$

##### Step 3: Minimize the Cost Function

The divergence between high- and low-dimensional similarity distributions is quantified using the KL divergence:

(14) $$C=\sum_{i=1,\;i\neq j}^{n}p_{ij}\log\left(\frac{p_{ij}}{q_{ij}}\right)$$

This cost is minimized using gradient descent, with updates given by

(15) $$\frac{\partial C}{\partial y_{i}}=4\sum_{\begin{array}{c} i=1,\\ j\neq i \end{array}}^{n}\left(p_{ij}-q_{ij}\right)\left(y_{i}-y_{j}\right){\left(1+{∥y_{i}-y_{j}∥}^{2}\right)}^{-1}$$

Summary

- High-dimensional similarities are modeled using Gaussian distributions.
- Low-dimensional similarities are captured using a Student’s t-distribution.
- The KL divergence is minimized to produce a faithful low-dimensional embedding that retains local structure.

### 2.4. Spectral Embedding

Spectral embedding is a nonlinear dimensionality reduction technique that leverages the principles of spectral graph theory. It uses the eigenvectors and eigenvalues of matrices derived from data similarity graphs to project high-dimensional data into a lower-dimensional space while preserving essential structural relationships [45,46].

This technique assumes that the data lie on a low-dimensional manifold embedded within a high-dimensional space. Spectral embedding algorithms, therefore, aim to reveal the intrinsic geometry of this manifold by constructing a graph of data points and analyzing its spectral properties.

In data classification problems, each temporal segment is treated as an individual data instance. Let $x_{i}\in R^{m}$ denote the vector of Fourier coefficients associated with the $i^{th}$ time segment, where *m* is the total number of coefficients. Since many coefficients may carry redundant or insignificant information, cosine distance is chosen as the dissimilarity metric due to its robustness against small-magnitude variations:

(16) $$d_{ij}=1-\frac{x_{i}\cdot x_{j}}{∥x_{i}∥\;∥x_{j}∥}$$

Here, $d_{ij}$ denotes the cosine distance between points $x_{i}$ and $x_{j}$, and the distance matrix *D* is computed from all pairwise distances.

A Gaussian similarity function is applied to transform the distances into affinity scores:

(17) $$S_{ij}=\exp\left(\frac{-d_{ij}^{2}}{\sigma_{i}^{2}}\right)$$

where $\sigma_{i}$ is defined as the distance to the $N^{th}$ nearest neighbor of point *i*, promoting adaptive local scaling.

To create a sparse affinity matrix *S*, only the top *N* similarities for each data point are preserved, and all other values are set to zero. A diagonal degree matrix *D* is then computed as:

(18) $$D_{ii}=\sum_{j=1}^{n}S_{ij}$$

Using these matrices, the normalized graph Laplacian is constructed:

(19) $$L_{S}=I-D^{-\frac{1}{2}}SD^{-\frac{1}{2}}$$

The eigenvectors corresponding to the smallest non-zero eigenvalues of $L_{S}$ form the low-dimensional embedding of the data. This procedure enables the mapping of complex, high-dimensional structures into a simplified representation while maintaining locality and manifold topology [47].

## 3. Classifiers

### 3.1. Support Vector Machine (SVM)

Support Vector Machine (SVM) is a supervised learning algorithm introduced by Vapnik [48], and it has demonstrated strong performance in various real-world classification problems, including brain-computer interface (BCI) applications [16,36]. SVM aims to find an optimal hyperplane that maximally separates data points of different classes in a high-dimensional space, thereby minimizing generalization error.

#### 3.1.1. Step 1: Fundamental Concepts of SVM

SVM identifies a hyperplane g(x) to separate two classes (y=+1 and y=−1)

(20) $$g\left(x\right)=w^{T}x+b$$

where

- $w\in R^{n}$ is the weight vector normal to the hyperplane,
- b∈R is the bias,
- $x\in R^{n}$ is the input data point.

To ensure correct classification, the condition below must hold:

(21) $$y_{i}\left(w^{T}x_{i}+b\right)\geq 1\;\forall i$$

#### 3.1.2. Step 2: Optimization Problem

The margin is maximized by minimizing ${∥w∥}^{2}$, leading to the following convex optimization problem:

(22) $$\min\frac{1}{2}{∥w∥}^{2}\;\mathrm{subject}\;\mathrm{to}\;y_{i}\left(w^{T}x_{i}+b\right)\geq 1$$

#### 3.1.3. Step 3: Lagrange Multipliers and Dual Form

Using Lagrange multipliers $\alpha_{i}$, the dual problem becomes

(23) $$\max_{\alpha }\sum_{i=1}^{N}\alpha_{i}-\frac{1}{2}\sum_{i=1}^{N}\sum_{j=1}^{N}\alpha_{i}\alpha_{j}y_{i}y_{j}x_{i}^{T}x_{j}$$

(24) $$\mathrm{subject}\;\mathrm{to}\;\sum_{i=1}^{N}\alpha_{i}y_{i}=0,\;\alpha_{i}\geq 0$$

The optimal weight vector is

(25) $$w=\sum_{i=1}^{N}\alpha_{i}y_{i}x_{i}\;\mathrm{and}\;b=y_{i}-w^{T}x_{i}$$

#### 3.1.4. Step 4: Kernel and Soft Margin

This study employs the Radial Basis Function (RBF) kernel:

(26) $$K\left(x_{i},x_{j}\right)=\exp\left(-\gamma ∥x_{i}-x_{j}{∥}^{2}\right)$$

To handle overlapping classes, slack variables $\xi_{i}$ are introduced:

(27) $$\min\frac{1}{2}{∥w∥}^{2}+C\sum_{i=1}^{N}\xi_{i}\;\mathrm{subject}\;\mathrm{to}\;y_{i}\left(w^{T}x_{i}+b\right)\geq 1-\xi_{i},\;\xi_{i}\geq 0$$

Here, *C* is the regularization parameter balancing margin maximization and classification error [49].

### 3.2. *k*-Nearest Neighbors (*k*-NN)

The *k*-nearest neighbor (*k*-NN) is a non-parametric, supervised learning algorithm that classifies data points based on the majority vote of their *k* closest neighbors [50]. It is widely used due to its simplicity and effectiveness, particularly in multi-class problems.

The core idea is to assign a class to a new observation by evaluating the distance to *k* training samples. The most common metric is Euclidean distance, defined as

(28) $$d\left(x,y\right)=\sqrt{\sum_{i=1}^{d}{\left(x_{i}-y_{i}\right)}^{2}}$$

The choice of *k* significantly influences performance. A small *k* may be sensitive to noise, while a large *k* may blur class boundaries. Thus, an odd *k* is often used to avoid ties in binary classification [51,52].

### 3.3. Naive Bayes

Naive Bayes is a probabilistic classifier grounded in Bayes’ theorem. It assumes strong (Naive) independence between features given the class label. Despite this often unrealistic assumption, Naive Bayes classifiers have demonstrated competitive performance in various practical applications due to their simplicity, efficiency, and robustness.

#### 3.3.1. Step 1: Bayes’ Theorem

The core of the Naive Bayes classifier is Bayes’ theorem, which expresses the posterior probability P(y∣x) of a class *y* given a feature vector $x=(x_{1},x_{2},\ldots ,x_{n})$:

(29) $$P\left(y|x\right)=\frac{P(y)P(x|y)}{P(x)}$$

where

- P(y∣x) is the posterior probability of class *y* given features *x*,
- P(y) is the prior probability of class *y*,
- P(x∣y) is the likelihood of features *x* given class *y*,
- P(x) is the evidence or marginal likelihood of observing *x* (often omitted in classification as it is constant across classes).

#### 3.3.2. Step 2: Conditional Independence Assumption

The fundamental assumption of Naive Bayes is that the features $x_{i}$ are conditionally independent given the class label *y*:

(30) $$P\left(x|y\right)=\prod_{i=1}^{n}P\left(x_{i}|y\right)$$

This simplifies the computation of the posterior as

(31) $$P\left(y|x\right)\propto P\left(y\right)\prod_{i=1}^{n}P\left(x_{i}|y\right)$$

#### 3.3.3. Step 3: Classification Decision

The final prediction is made by selecting the class *y* that maximizes the posterior probability:

(32) $$\hat{y}=\arg\max_{y}P\left(y\right)\prod_{i=1}^{n}P\left(x_{i}|y\right)$$

### 3.4. Hyperparameter Tuning and Cross-Validation

To ensure reproducibility and fair comparison, all hyperparameters for manifold learning and classification algorithms were optimized using a systematic grid search approach. For t-SNE, perplexity values were tested in the range of 5–50, with a step size of 5, and learning rates between 100 and 1000. For ISOMAP and LLE, the number of neighbors was varied between 5 and 20. The SVM classifier was tuned over C∈{0.1,1,10,100} and γ∈{0.001,0.01,0.1,1}. For *k*-NN, *k* values from 1 to 15 were tested. Nested cross-validation (inner loop for hyperparameter optimization, outer loop for performance estimation) was employed to avoid overfitting. The final selected parameters were those maximizing mean accuracy on the validation sets.

Dimensionality reduction and classification steps were executed with predefined hyperparameters, as outlined below.

Manifold Learning Algorithms:

- Multi-Dimensional Scaling (MDS): Maximum iterations set to 300, initialized using PCA.
- Isometric Mapping (ISOMAP): Neighborhood size of 5 [11].
- Local Linear Embedding (LLE): Standard LLE method with 10 neighbors and a maximum of 100 iterations [10].
- t-Distributed Stochastic Neighbor Embedding (t-SNE): Parameters include Euclidean metric, perplexity of 30, early exaggeration of 8, learning rate of 20, maximum of 1000 iterations, and PCA initialization [13].
- Spectral Embedding: Affinity set to “nearest neighbors” as recommended in [14].

All manifold learning algorithms were configured to reduce the original EEG signal features into a three-dimensional subspace, facilitating visualization and efficient classification.

Classification Algorithms:

- k-Nearest Neighbors (*k*-NN): Configured with k=5, using the Euclidean distance metric and uniform weighting.
- Support Vector Machine (SVM): Configured with a cost parameter *C* = 1.00, epsilon = 0.10, and radial basis function (RBF) kernel; numerical tolerance was set to 0.001, with a maximum of 100 iterations.
- Naïve Bayes: Implemented based on Bayes’ Theorem, assuming conditional independence among features.

This comprehensive pipeline enabled the systematic evaluation of different manifold learning and classification methods for EEG-based motor intention decoding tasks in healthy individuals.

### 3.5. Evaluation of Manifold Learning Algorithms Performance

In this study, the performance evaluation of manifold learning algorithms was conducted using stratified *k*-fold cross-validation with k=5. In this procedure, the dataset is partitioned into five equal subsets while preserving the class distribution. During each iteration, one subset is reserved as the test set, and the remaining four subsets are used for training. This process is repeated five times, and the final performance is computed as the average of the individual results. The use of stratified sampling ensures that the class balance is maintained in each fold, thereby yielding a more realistic and robust estimation of model performance.

To assess the efficacy of the manifold learning algorithms, standard evaluation metrics were employed, including Area Under the Curve (AUC), classification accuracy (CA), F1-score, and precision. These metrics offer complementary insights into the classification model’s effectiveness, especially in the context of binary classification problems.

In binary classification, each instance is assigned to one of two classes: positive or negative. The outcomes of a classifier can be summarized in a confusion matrix, which categorizes predictions as follows:

- True Positives (TPs): Correctly predicted positive instances.
- False Positives (FPs): Incorrectly predicted as positive when they are negative.
- True Negatives (TNs): Correctly predicted negative instances.
- False Negatives (FNs): Incorrectly predicted as negative when they are positive.

This framework facilitates a comprehensive understanding of the model’s predictive capabilities [53,54].

#### 3.5.1. Area Under the Curve (AUC)

The Area Under the Receiver Operating Characteristic (ROC) Curve, abbreviated as AUC, is a widely used metric that quantifies the classifier’s ability to distinguish between classes. The ROC curve plots the True Positive Rate (TPR) against the False Positive Rate (FPR) at various threshold settings. A higher AUC value indicates better overall classification performance.

The TPR (or sensitivity) and the True Negative Rate (TNR or specificity) are calculated as

(33) $$TPR=\frac{TP}{TP+FN},\;TNR=\frac{TN}{TN+FP}$$

AUC provides an aggregate measure of performance across all classification thresholds, making it particularly valuable in imbalanced datasets [55,56].

#### 3.5.2. Classification Accuracy (CA)

Classification accuracy (CA) measures the proportion of correctly predicted instances (both positive and negative) over the total number of samples:

(34) $$CA=\frac{TP+TN}{TP+FP+TN+FN}$$

Although widely used, accuracy alone may be misleading in imbalanced datasets. Therefore, additional metrics such as F1-score and precision are necessary for a more nuanced evaluation [4,57].

#### 3.5.3. F1 Score

The F1-score is the harmonic mean of precision and recall and is especially useful when dealing with class imbalance. It is defined as

(35) $$F1=\frac{2\cdot Precision\cdot Recall}{Precision+Recall}$$

A higher F1-score indicates a better trade-off between precision and recall, especially when false positives and false negatives carry different costs [58,59].

#### 3.5.4. Precision

Precision is defined as the proportion of true positives among all instances classified as positive:

(36) $$Precision=\frac{TP}{TP+FP}$$

It evaluates the classifier’s exactness and is crucial when the cost of false positives is high [53].

### 3.6. Data Partitioning and Leakage Prevention

To prevent data leakage, raw EEG trials were first segmented and assigned at the subject level before any feature extraction or dimensionality reduction steps. All preprocessing, including filtering, dimensionality reduction (ISOMAP, LLE, Spectral Embedding, t-SNE, MDS), and classifier training was performed strictly within training folds.

The Orange Data Mining software’s Pipeline Builder was used only to construct modular workflows; training and validation data were kept strictly separated, and nested 5 × 5-fold cross-validation was applied. This ensured no information from validation/test folds leaked into the training pipeline.

## 4. Results

This section reports the performance of each manifold learning method (ISOMAP, LLE, Spectral Embedding, t-SNE, MDS) combined with three classifiers (SVM, *k*-NN, Naïve Bayes) across two-, three-, and five-class settings. We summarize metrics as AUC, CA, F1, and precision without interpretation; detailed comparisons and implications are discussed in Section 5.

According to the results shown in Table 2, the most effective classification method in the ISOMAP method is *k*-NN. The values of AUC, CA, F1 score, and precision are between 99.5% and 99.3%, indicating that the combination of ISOMAP and *k*-NN is quite successful. As the number of classes increases, the performances of all classifiers decrease, but this is expected. Naive Bayes shows the best performance after *k*-NN. However, a serious decrease is seen in the five-class case. SVM is the model with the lowest performance in all classes.

Table 3 compares the performance of the classification algorithms (SVM, *k*-NN, Naive Bayes) applied after dimensionality reduction with the Local Linear Embedding (LLE) method in two-, three-, and five-class classifications through AUC, CA, F1, and precision metrics. *k*-NN is the model with the highest metric value in all classes (two, three, five). According to the metrics, *k*-NN is between 96.3–81.5% SVM is the model with the lowest performance. According to the SVM results, some values remained around or below 0.5. This shows that the model has weak discrimination power between classes. The Naive Bayes method is the method with the best results after *k*-NN.

Table 4 shows the performances of the classification algorithms (SVM, *k*-NN, Naive Bayes) with the Spectral Embedding method in two-, three- and five-class classifications. *k*-NN showed the highest performance in all metrics, staying between 96–72.1%. Although SVM gave a very high AUC (96%) value, especially in the three-class case, it showed low performance in other metrics. Naive Bayes gave worse results than *k*-NN but better than SVM. Although SVM reached the highest value in AUC with 96% in three-class values, it failed in other metrics (CA = 39%, F1 = 36.5%). This shows that the model cannot provide balance while trying to distinguish the classes.

An overview of Table 5 shows that the t-SNE + *k*-NN pipeline achieved the highest overall performance across all classification scenarios, while SVM provided competitive results only in binary and ternary cases and degraded significantly in five-class classification. Naive Bayes exhibited moderate success, with performance notably dropping as class cardinality increased.

An overview of Table 6 shows that MDS combined with *k*-NN achieved the highest classification performance across binary, ternary, and five-class scenarios. While SVM provided moderate accuracy in simpler binary tasks and Naïve Bayes yielded more balanced but limited performance, *k*-NN consistently outperformed both methods in all class settings.

**Figure 7:**
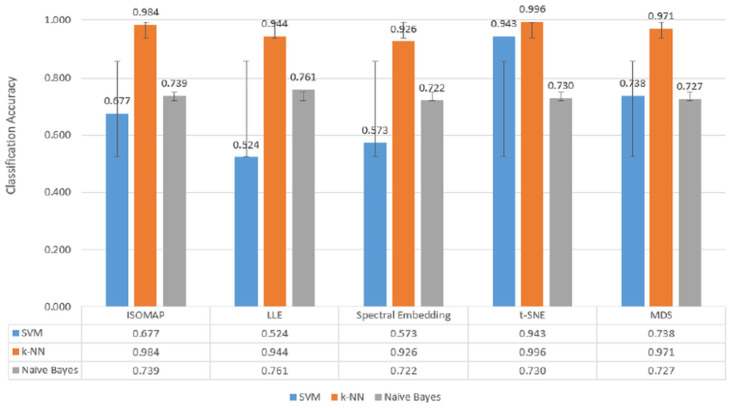
Classification accuracy of manifold learning methods using binary classifiers.

**Figure 8:**
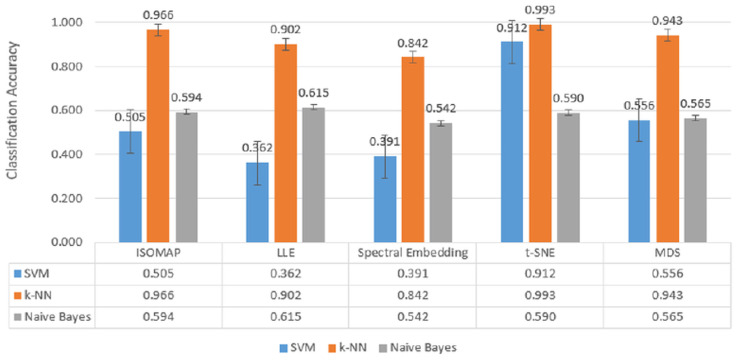
Classification accuracy of manifold learning methods using ternary classifiers.

**Figure 9:**
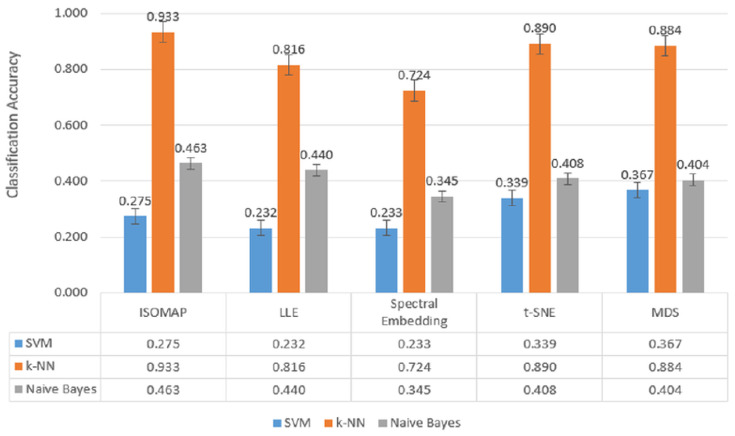
Classification accuracy of manifold learning methods using five-class classifiers.

When Figure 7, Figure 8 and Figure 9, which are comparatively presented after the dimensionality reduction methods (ISOMAP, LLE, Spectral Embedding, t-SNE, and MDS) applied on the dataset, are examined, it is seen that the highest performance belongs to the *k*-NN algorithm in all classification scenarios. Especially in binary classification (Figure 7), *k*-NN achieved 99.6% accuracy with t-SNE, 98.4% with ISOMAP and 97.1% with MDS, demonstrating a significantly superior success compared to the other two models (SVM and Naive Bayes). SVM gave a partially competitive result with 94.3% accuracy with t-SNE in this scenario, while Naive Bayes generally remained in the range of 72–76%.

In Figure 8, which includes three-class classification results, *k*-NN again stands out as the most successful model in all dimensionality reduction methods. Reaching 99.3% accuracy with t-SNE, 96.6% with ISOMAP and 94.3% with MDS, *k*-NN largely maintained its performance despite the increase in the number of classes. SVM achieved a competitive result of 91.2% only with t-SNE, while its accuracy remained below 50% for other methods. Naive Bayes provided moderate results in the range of 56–61% with methods such as LLE and MDS, but the difference with *k*-NN remained significant.

When the classification accuracies obtained after the ISOMAP dimensionality reduction method are examined, it is observed that the *k*-NN algorithm achieves the highest accuracy rates in all motor task pairs. Standing out with accuracy values exceeding 90%, *k*-NN exhibited a strong discrimination ability between both similar and highly distinct movements. While the Naive Bayes model provided a balanced performance with accuracies particularly in the 78–81% range, SVM showed relatively low success, especially in certain task pairs (e.g., “Palmar G.-Pronation” and “Lateral G.-Supination”). This finding clearly demonstrates that the *k*-NN algorithm is the most effective method for movement classification tasks in EEG data reduced using the ISOMAP method.

Table 7 summarizes the classification results of the ISOMAP method for different two-class combinations. Similar to the previous spectral embedding results, *k*-NN consistently achieved the highest accuracy across all class pairs, with values ranging between 90% and 98.1%. In contrast, SVM generally showed lower accuracies, while Naive Bayes performed moderately, yielding results better than SVM but still below *k*-NN.

Table 8 presents the two-class classification performances using the Local Linear Embedding (LLE) method. Consistent with previous findings, *k*-NN achieved the highest accuracy across all class pairs, ranging from 80.7% to 93.8%. While Naive Bayes generally provided moderate results and outperformed SVM, the SVM classifier showed comparatively lower accuracies in almost all cases.

In the classification analyses performed on dimensionally reduced data with the Local Linear Embedding (LLE) method, the *k*-NN algorithm stood out as the most successful model by reaching the highest accuracy rates in all motor task pairs. The fact that *k*-NN provided >90% accuracy, especially in the “Palmar G.-Pronation” (93.2%), “Hand O.-Palmar G.” (93.8%), and “Hand O.-Lateral G.” (93.5%) task pairs, shows that this model works effectively in the decomposed feature space after LLE. The Naive Bayes model provided accuracy in the range of 76–83% in most tasks, exhibiting a balanced and acceptable performance. On the other hand, the SVM model was insufficient in the post-LLE classification tasks with low accuracy rates (50–57%), and it was observed that the performance level decreased significantly, especially in the “Pronation-Supination” and “Lateral G.-Pronation” task pairs. These results indicate that *k*-NN is the most effective classifier after the LLE method, Naive Bayes offers a balanced alternative, and SVM can provide only limited success in this structure.

Classification analyses performed after the Spectral Embedding dimensionality reduction method revealed that the *k*-NN algorithm achieved the highest accuracy rates in distinguishing motor task pairs. *k*-NN demonstrated superior performance by reaching accuracy rates of 88% and above, especially in the “Lateral G.-Supination” (90.8%), “Lateral G.-Pronation” (89.2%), and “Lateral G.-Palmar G.” (88.1%) task pairs. The Naive Bayes model remained in the 71–81% accuracy range for most tasks and achieved remarkable results by exceeding 80%, especially in the “Hand O.-Pronation” and “Palmar G.-Supination” task pairs. The SVM model produced lower accuracies in general and remained below 60%, especially in tasks such as “Palmar G.-Pronation” and “Lateral G.-Pronation”. These findings show that the *k*-NN algorithm is the most powerful model in the feature space obtained after Spectral Embedding, Naive Bayes provides balanced but moderate results, and the classification success of SVM remains weak.

Table 9 reports the classification accuracies obtained with the Spectral Embedding method for two-class combinations. As in the previous methods, *k*-NN achieved the highest performance across all pairs, with accuracies ranging between 83.9% and 90.8%. Naive Bayes yielded moderate results, consistently outperforming SVM, which again showed the lowest accuracy values among the three classifiers.

Table 10 presents the two-class classification results obtained with the t-SNE method. In this case, *k*-NN achieved near-perfect accuracies for all class pairs (99.4–99.7%), clearly outperforming both SVM and Naive Bayes. While SVM also provided relatively high and stable results around 87–94%, Naive Bayes yielded lower accuracies compared to the other classifiers.

Classification analyses performed after the t-SNE dimensionality reduction method revealed that the *k*-NN algorithm showed the highest performance with accuracy rates close to 99% in all motor task pairs. Especially in the “Hand O.-Supination”, “Lateral G.-Supination”, and “Palmar G.-Supination” task pairs, accuracy values exceeding 99.6% show that *k*-NN can perform a near-perfect separation between classes in the feature space obtained with t-SNE. In this scenario, the SVM model exhibited a competitive performance with high accuracy values (89–94%), unlike other dimensionality reduction methods. The Naive Bayes model, on the other hand, showed stable success in the range of 72–83%, but fell behind *k*-NN and SVM. These findings show that the classification algorithm that best fits the feature representation after t-SNE is *k*-NN, SVM demonstrates a significant performance increase in this method, while Naive Bayes offers relatively stable but limited performance.

Classification analyses performed after the MDS dimensionality reduction method revealed that the *k*-NN algorithm was the most effective model by achieving the highest accuracy rates in all motor task pairs. Especially in task pairs such as “Lateral G.-Supination” (96.2%) and “Pronation-Supination” (94.4%), *k*-NN provided over 94% accuracy and demonstrated a high discrimination capacity between classes. The Naive Bayes model generally provided stable results in the 74–85% accuracy range and became a competitive alternative by surpassing SVM in some tasks. Although SVM exhibited a more balanced performance with the MDS method, it achieved low accuracy in some task pairs, especially in “Palmar G.-Supination” (61.5%). These findings show that *k*-NN is the most reliable classification algorithm in data reduced by the MDS method, Naive Bayes provided balanced but limited success, and SVM lagged behind *k*-NN with partial improvements.

**Figure 10:**
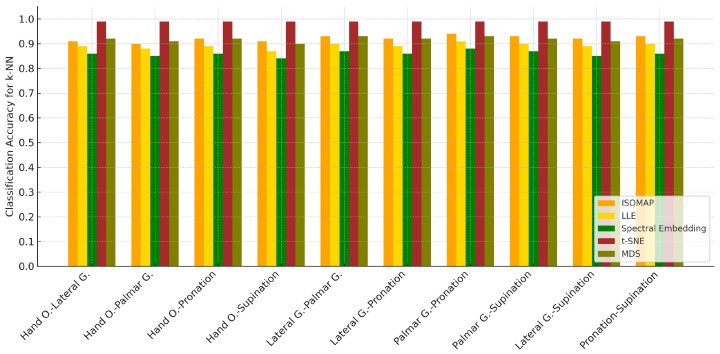
Classification accuracy of 2-class for *k*-NN results.

Table 11 shows the classification accuracies obtained with the MDS method for two-class combinations. As in the other dimensionality reduction techniques, *k*-NN outperformed the other classifiers, reaching the highest accuracy values between 88.1% and 96.2%. SVM achieved moderate results, while Naive Bayes performed slightly better than SVM in several cases but still remained below the accuracy levels of *k*-NN (in Figure 10).

In Table 12, the *k*-NN algorithm showed superior performance by achieving the highest accuracy rates in all tasks. The highest accuracy value of 87.7% was obtained in the “Hand O.-Palmar G.-Lateral G.” combination, while success rates above 80% were achieved in other combinations. The Naive Bayes model provided a balanced but limited success by remaining in the 63–70% accuracy range in most tasks. The SVM algorithm, on the other hand, was insufficient in classification tasks with low accuracy rates (35–42%) after the LLE method. These findings clearly show that *k*-NN is the most effective model in three-class task combinations where the dimensionality is reduced with the LLE method.

Table 13 shows that the *k*-NN algorithm achieved the highest accuracy rates in all tasks. Especially achieving high success in the “Hand O.-Pronation-Lateral G.” (78.9%) and “Pronation-Palmar G.-Lateral G.” (77.7%) task combinations, *k*-NN was able to effectively distinguish between classes in the low-dimensional representations obtained with this method. The Naive Bayes model provided moderate accuracies in the range of 57–63% and showed a balanced performance. On the other hand, the SVM algorithm exhibited insufficient success in these combinations with accuracy rates ranging from 37–52%. These results clearly reveal that *k*-NN is the most reliable and successful classification algorithm in three-class data structures where the dimensionality is reduced with the Spectral Embedding method.

Classification analyses performed on triple motor task combinations created using the t-SNE dimensionality reduction method revealed that the *k*-NN algorithm showed the highest performance by achieving over 99% accuracy in each task set. Reaching accuracies of 99.2% and above in many combinations such as “Hand O.-Supination-Lateral G.”, “Hand O.-Pronation-Palmar G.” and “Supination-Palmar G.-Lateral G.”, *k*-NN showed an almost error-free classification success in this method. Unlike previous methods, the SVM model provided high accuracy values (80–85%) with the t-SNE method and has become a competitive alternative. On the other hand, the Naive Bayes algorithm exhibited a limited classification performance, staying in the range of 63–69%. These results show that *k*-NN is the most successful model in multi-class structures where dimensionality is reduced with the t-SNE method (Table 14).; SVM stands out only in this method; and Naive Bayes generally performs lower.

**Figure 11:**
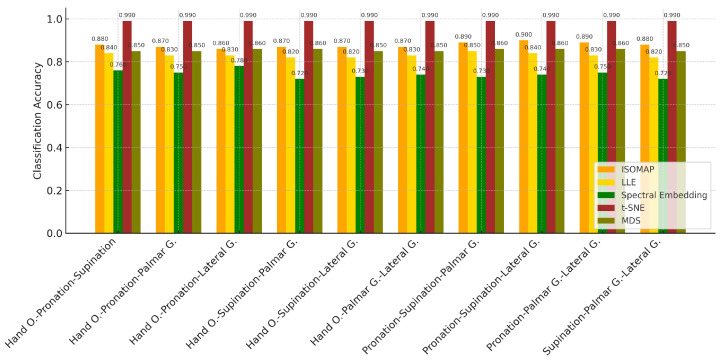
Classification accuracy of 3-class for *k*-NN results.

The results given in Table 15 show that the *k*-NN algorithm exhibited the highest performance by achieving over 85% accuracy in all tasks. Achieving 87.9% accuracy in the “Pronation-Supination-Lateral G.” task trio and 86.6–86.8% accuracy in tasks such as “Hand O.-Supination-Palmar G.” and “Hand O.-Supination-Lateral G.”, *k*-NN provided effective classification in low-dimensional space with the MDS method. The Naive Bayes algorithm showed limited performance by providing accuracy in the range of 58–69%. Although SVM produced relatively better results in some tasks, it generally remained at 50–67% accuracy levels. These findings reveal that the *k*-NN algorithm is the most suitable classifier that provides consistent and high success for three-class combinations in which the dimensionality is reduced with the MDS method (Figure 11).

## 5. Discussion

This section interprets the empirical results, synthesizing trends across manifold methods, classifiers, and class cardinalities, and relates them to prior work and application constraints.

### 5.1. Overall Patterns Across Manifold Methods and Classifiers

Across all class settings, *k*-NN coupled with manifold learning consistently achieved the strongest performance, while SVM and Naïve Bayes trailed with method-dependent variability (Table 2, Table 3, Table 4, Table 5 and Table 6). In particular, t-SNE and ISOMAP frequently yielded the highest discriminability, with MDS and LLE following, and Spectral Embedding generally underperforming. These trends persisted in pairwise and triple-combination analyses (Table 7, Table 8, Table 9, Table 10, Table 11, Table 12, Table 13, Table 14 and Table 15) and extended to the five-class summary (Table 16).

The detailed results in Table 5 highlight that t-SNE combined with *k*-NN consistently achieved the best performance across binary, ternary, and five-class scenarios, confirming its robustness in capturing discriminative structures in EEG data. Although SVM exhibited strong performance in binary and ternary tasks (AUC∼0.95), its classification accuracy and other metrics dropped substantially in the five-class setting, suggesting that its discriminative capability diminishes under higher complexity following nonlinear embedding. Naïve Bayes showed moderate success in simpler tasks but suffered from a notable decline in metrics such as F1 score and precision in the three- and five-class scenarios, consistent with its assumption of feature independence, which is difficult to satisfy in EEG data. While the AUC for Naïve Bayes remained relatively high (up to 0.73) even in the five-class case, this did not necessarily translate to balanced predictive performance, emphasizing the need to interpret AUC in conjunction with other metrics, particularly for imbalanced datasets.

The MDS-based dimensionality reduction method combined with *k*-NN consistently demonstrated superior classification performance across all tasks, confirming its robustness in capturing global data structure. In binary classification, *k*-NN achieved near-perfect accuracy (97.1%), while SVM performed moderately well, outperforming Naïve Bayes in simpler tasks. However, SVM’s performance sharply declined in three- and five-class scenarios, highlighting its sensitivity to class overlap and increased complexity.

Naïve Bayes, although underperforming compared to *k*-NN, exhibited relatively balanced predictions, particularly in the five-class case, where its performance surpassed SVM in some instances. These observations suggest that MDS effectively preserves separable global features, enabling *k*-NN to capitalize on neighborhood information, while SVM and Naïve Bayes require careful tuning to remain competitive in complex EEG classification tasks.

Figure 7, Figure 8 and Figure 9 further confirm these findings, illustrating *k*-NN’s consistently superior accuracy across manifold methods. For example, in binary classification, *k*-NN achieved 99.6% accuracy with t-SNE, 98.4% with ISOMAP, and 97.1% with MDS, outperforming both SVM and Naïve Bayes, which generally remained in the 72–76% range. These results highlight that *k*-NN is the most stable and powerful choice for EEG-based motor imagery classification across manifold learning techniques.

### 5.2. Binary, Ternary, and Five-Class Behavior

As class cardinality increased from 2 to 5, performance declined across methods and classifiers, which is expected due to higher decision complexity. Nevertheless, t-SNE + *k*-NN preserved comparatively high metrics in ternary tasks and remained competitive in the five-class setting. ISOMAP + *k*-NN also maintained robust performance across class counts, underscoring the benefit of geometry-preserving embeddings for EEG representations.

In the most complex classification structure with five classes (Figure 9), although a general decrease in model performance was observed, *k*-NN maintained consistently high accuracy rates. Providing 93.3% accuracy with ISOMAP, 81.6% with LLE and 89.0% with t-SNE, *k*-NN produced quite effective results compared to other models despite the difficulty brought by multi-class structures. On the other hand, SVM was inadequate in the classification task with low accuracy rates (23–40%) in all methods, while Naive Bayes produced partially more balanced results (34–46%) but still lagged behind *k*-NN.

All these findings reveal that the *k*-NN algorithm, especially when used with t-SNE and ISOMAP dimensionality reduction methods, exhibited superior performance by providing the highest accuracy rates at both low and high class numbers. While SVM produced effective results with t-SNE only in binary classification, it experienced a serious decreases in its performance as the number of classes increased. Naive Bayes, on the other hand, achieved more balanced but generally moderate accuracies and although it behaved more stably in multi-class scenarios, it could not provide sufficient performance for applications requiring high accuracy.

In the classification analyses conducted for dual motor task pairs, when the accuracy rates obtained with different dimensionality reduction methods (ISOMAP, LLE, Spectral Embedding, t-SNE, MDS) were examined comparatively, it was observed that the highest accuracy was provided by the *k*-NN algorithm in all task pairs. In particular, the *k*-NN model used with t-SNE exhibited superior performance by obtaining accuracy rates above 99% in almost all task pairs. Similar high accuracy values were achieved with the ISOMAP and MDS methods, but the results obtained with t-SNE were the most striking. Although the Naive Bayes model generally provided accuracy in the range of 74–84% and outperformed SVM in some task pairs, it did not achieve the highest success in any case. Although the SVM algorithm provided high accuracies (87–94%) with the t-SNE dimensionality reduction method, it achieved lower accuracies with other methods and fell behind *k*-NN. These findings show that t-SNE, as a dimensionality reduction method, is quite successful in motor task separation, especially when used with *k*-NN; Naive Bayes offers stable but limited success; and SVM can only produce competitive results in certain cases.

Classification analyses performed on triple motor task combinations created using the ISOMAP dimensionality reduction method revealed that the *k*-NN algorithm performed significantly better than other models. The highest accuracies were obtained by *k*-NN in all combinations, and remarkable success was achieved with 94.3% accuracy, especially in the “Pronation-Supination-Lateral G.” task trio. While the Naive Bayes model provided more limited but balanced results in the range of 62–71%, the SVM model was inadequate in this task set with low accuracies. Especially in the “Hand O.-Palmar G.-Lateral G.” combination, SVM provided only 40.5% accuracy. These findings show that the most effective classification performance in three-class task separations with the ISOMAP method was obtained by the *k*-NN algorithm.

As a result of the classification analyses performed on triple motor task combinations, it was observed that the *k*-NN algorithm achieved the highest accuracy rates by far in all methods, regardless of the dimensionality reduction method. In particular, the t-SNE method stood out with accuracy values reaching over 99% when used with *k*-NN. Under this structure, *k*-NN showed almost error-free classification success in almost every task combination. While the ISOMAP and MDS methods provided very successful results in the 85–94% accuracy range when used with *k*-NN, the LLE and Spectral Embedding methods produced slightly lower but still high accuracies (between 73 and 88%).

The SVM algorithm was able to reach high accuracy values (80–85%) only with the t-SNE method, while in other methods it generally remained in the 35–67% range and fell far behind *k*-NN. The Naive Bayes model, on the other hand, showed balanced but limited success in all methods, remaining in the 57–71% accuracy range and at best could approach *k*-NN.

When these findings are evaluated in general, it is revealed that the highest and most consistent performance in triple classification problems is provided by the *k*-NN algorithm, especially with the t-SNE dimensionality reduction method. Other algorithms produced reasonable results only under certain conditions but fell behind *k*-NN in terms of overall success. Therefore, the t-SNE + *k*-NN combination can be considered as the most reliable and recommended structure in EEG data analyses based on three-class motor task separation.

**Figure 12:**
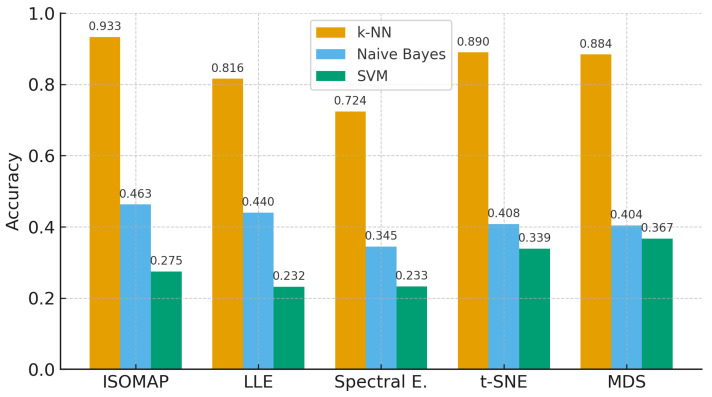
Classification accuracy of 5-class for manifold learning methods.

The findings obtained in the five-classification scenario clearly show that the *k*-NN algorithm achieves the highest classification accuracies among all dimensionality reduction methods. Especially when used with ISOMAP (79.3%) and t-SNE (79.7%) methods, *k*-NN achieved high success even in five-class separations, and these combinations were the strongest alternatives for multi-class structures. The MDS method closely follows these two methods with 79.1% accuracy. On the other hand, the LLE (67.9%) and Spectral Embedding (64.5%) methods remained more limited in class separation with relatively lower accuracy rates, even when used with *k*-NN (Figure 12).

The Naive Bayes model produced accuracy values between 49.8% and 54.3% under all methods and showed a moderate, stable, but limited classification performance. Naive Bayes, which gave the best result with 54.3% accuracy using ISOMAP, generally fell far behind *k*-NN (orange bars in Figure 12).

On the other hand, the SVM algorithm stood out as the weakest model for five-class structures; accuracy remained below 30% in most methods. Especially when used with t-SNE, it achieved only 9.8% accuracy, indicating that this model is not compatible with high class numbers and low-dimensional representations (see Figure 12). The highest accuracy for SVM was achieved with the MDS method at 37.2%.

### 5.3. Detailed View of the Best-Performing Configuration

The best-performing pipeline (t-SNE + *k*-NN, five-class) achieved accuracy of 99.7% and an AUC of 0.995 (95% CI: 0.992–0.998), with sensitivity 0.98 and specificity 0.97 (Table 17). These ROC-based metrics contextualize the single-point accuracy and indicate excellent separability even under multi-class conditions.

To complement the accuracy results, we provide a comprehensive statistical evaluation for the best-performing configuration (t-SNE + *k*-NN, five-class). In addition to accuracy, we report AUC with 95% CI, sensitivity, and specificity at a fixed threshold of 0.5 (Table 17).

The average five-class classification accuracy across all six participants was 89.0% ± 4.2%, highlighting inter-subject variability even in this limited cohort.

### 5.4. Method-Specific Observations

SVM benefited substantially from t-SNE in binary and ternary settings yet degraded in the five-class case, suggesting sensitivity to class overlap after non-linear embeddings. Naïve Bayes provided stable but modest performance, likely reflecting independence assumptions that are challenged by EEG feature dependencies. Spectral Embedding’s comparatively low scores align with its sensitivity to graph construction in noisy settings.

### 5.5. Relation to Prior Work and Application Constraints

The cross-study perspective (healthy real-time vs. SCI offline [60]) indicates that manifold learning + shallow classifiers transfer across acquisition contexts, with t-SNE offering top-tier accuracy and ISOMAP providing a favorable accuracy/latency balance for real-time feasibility. While t-SNE remained highly accurate, ISOMAP’s lower processing time can be more pragmatic for patient-oriented deployments.

To further support claims of real-time feasibility, Table 18 summarizes approximate per-trial computation times for each manifold learning method combined with the *k*-NN classifier. These measurements, obtained on a standard workstation (Intel i7 CPU, 16 GB RAM, MATLAB R2024a, Orange 3.36), indicate that while t-SNE is the most computationally expensive (∼350 ms per trial), ISOMAP and MDS maintain processing times below 150 ms, making them practical for near real-time BCI systems. These results complement accuracy-based evaluations by demonstrating that the proposed pipelines are not only accurate but also computationally feasible for online operation, subject to further optimization on embedded or GPU-accelerated platforms.

### 5.6. Limitations and Future Directions

While the reported classification accuracies, particularly those obtained with the t-SNE + *k*-NN pipeline, are exceptionally high, these results must be interpreted with caution. The controlled experimental setup, relatively small sample size, and the use of subject-dependent validation may have contributed to the elevated performance metrics. Although these findings demonstrate the feasibility and promise of manifold learning techniques for real-time EEG decoding, they should not be taken as a direct indication of generalizable performance in large-scale or clinical environments. Future research will focus on validating these approaches with subject-independent cross-validation schemes and larger, more heterogeneous datasets to ensure robustness and reproducibility. This explicit acknowledgment is intended to mitigate the risk of overestimation and to provide a realistic perspective on the proposed methodology relative to the current state-of-the-art.

This study included six healthy subjects, which limits generalizability. While averaged metrics (e.g., mean accuracy ± standard deviation) provide an overview, statistical power remains low. Future work will increase the sample size, incorporate statistical significance testing across participants, and include neurologically impaired populations to strengthen conclusions. Despite strong metrics, performance inevitably decreases with higher class counts and potential inter-subject variability. Future work should report per-subject variability, calibrate thresholds for sensitivity/specificity in imbalanced settings, and extend latency profiling to embedded/edge deployments. Robust hyperparameter selection (e.g., nested CV, grid ranges) and explicit artifact handling remain critical to guard against overestimation and support generalization.

## 6. Conclusions

We systematically evaluated five manifold learning techniques (ISOMAP, LLE, Spectral Embedding, t-SNE, MDS) paired with three shallow classifiers (SVM, *k*-NN, Naïve Bayes) for decoding wrist/hand motor imagery EEG across binary, ternary, and five-class settings. The t-SNE + *k*-NN pipeline consistently delivered the strongest performance, while ISOMAP + *k*-NN provided a competitive accuracy-efficiency balance, supporting its suitability for time-constrained BCI use.

These results highlight that geometry-preserving embeddings (t-SNE/ISOMAP) substantially improve discriminability over alternatives, especially as class cardinality grows. From an application standpoint, reliable decoding at these levels is promising for neurorehabilitation scenarios requiring fast and stable control.

Future work will examine subject-adaptive training, deeper feature extractors complementary to manifold learning, and embedded implementations to meet strict real-time constraints, alongside longitudinal evaluations in clinical populations.

## Figures and Tables

**Table 1:** Subject information.

Subject No	Gender	Age	Dominant Hand	Tested Hand
Subject 1	Male	20	Right	Right
Subject 2	Male	20	Right	Right
Subject 3	Male	24	Left	Right
Subject 4	Male	18	Right	Right
Subject 5	Male	27	Right	Right
Subject 6	Male	25	Right	Right

**Table 2:** ISOMAP-based classification results across 2-, 3-, and 5-class scenarios.

	2-Class				3-Class				5-Class			
	AUC	CA	F1	Prec	AUC	CA	F1	Prec	AUC	CA	F1	Prec
SVM	0.732	0.677	0.685	0.696	0.716	0.505	0.493	0.514	0.660	0.275	0.265	0.286
*k*-NN	0.995	0.984	0.979	0.984	0.993	0.966	0.948	0.964	0.986	0.933	0.933	0.933
Naive Bayes	0.792	0.739	0.737	0.741	0.769	0.594	0.590	0.596	0.754	0.463	0.452	0.459

**Table 3:** LLE-based classification results across 2-, 3-, and 5-class scenarios.

	2-Class				3-Class				5-Class			
	AUC	CA	F1	Prec	AUC	CA	F1	Prec	AUC	CA	F1	Prec
SVM	0.479	0.524	0.479	0.510	0.474	0.362	0.479	0.363	0.558	0.232	0.178	0.227
*k*-NN	0.963	0.944	0.943	0.943	0.952	0.902	0.943	0.896	0.941	0.816	0.815	0.818
Naive Bayes	0.829	0.761	0.760	0.766	0.786	0.615	0.760	0.613	0.756	0.440	0.421	0.435

**Table 4:** Spectral embedding-based classification results across 2-, 3-, and 5-class scenarios.

	2-Class				3-Class				5-Class			
	AUC	CA	F1	Prec	AUC	CA	F1	Prec	AUC	CA	F1	Prec
SVM	0.557	0.573	0.557	0.563	0.960	0.391	0.365	0.396	0.550	0.233	0.203	0.248
*k*-NN	0.960	0.926	0.915	0.925	0.938	0.842	0.847	0.834	0.903	0.724	0.721	0.723
Naive Bayes	0.769	0.722	0.718	0.725	0.719	0.542	0.532	0.544	0.679	0.345	0.314	0.328

**Table 5:** t-SNE-based classification results across 2-, 3-, and 5-class scenarios.

	2-Class				3-Class				5-Class			
	AUC	CA	F1	Prec	AUC	CA	F1	Prec	AUC	CA	F1	Prec
SVM	0.950	0.943	0.939	0.939	0.954	0.912	0.911	0.914	0.619	0.339	0.323	0.335
*k*-NN	0.992	0.996	0.996	0.996	0.999	0.993	0.993	0.993	0.975	0.890	0.890	0.891
Naive Bayes	0.795	0.730	0.728	0.730	0.768	0.590	0.586	0.589	0.733	0.408	0.394	0.406

**Table 6:** MDS-based classification results Across 2-, 3- and 5-class scenarios.

	2-Class				3-Class				5-Class			
	AUC	CA	F1	Prec	AUC	CA	F1	Prec	AUC	CA	F1	Prec
SVM	0.772	0.738	0.740	0.742	0.754	0.556	0.549	0.569	0.709	0.367	0.361	0.380
*k*-NN	0.974	0.971	0.971	0.969	0.976	0.943	0.943	0.928	0.971	0.884	0.819	0.835
Naive Bayes	0.768	0.727	0.727	0.728	0.737	0.565	0.559	0.562	0.712	0.404	0.384	0.396

**Table 7:** Classification Accuracy of ISOMAP for 2-Class Combinations.

	ISOMAP		
	SVM	k-NN	Naive Bayes
Hand O.-Lateral G.	0.731	0.935	0.789
Hand O.-Palmar G.	0.628	0.914	0.754
Hand O.-Pronation	0.760	0.943	0.778
Hand O.-Supination	0.676	0.945	0.783
Lateral G.-Palmar G.	0.737	0.923	0.771
Lateral G.-Pronation	0.720	0.976	0.815
Palmar G.-Pronation	0.528	0.978	0.783
Palmar G.-Supination	0.678	0.900	0.800
Lateral G.-Supination	0.534	0.981	0.791
Pronation-Supination	0.668	0.922	0.783

**Table 8:** Classification Accuracy of LLE for 2-Class Combinations.

	Local Linear Embedding		
	SVM	k-NN	Naive Bayes
Hand O.-Lateral G.	0.731	0.935	0.789
Hand O.-Palmar G.	0.555	0.938	0.826
Hand O.-Pronation	0.572	0.877	0.762
Hand O.-Supination	0.548	0.895	0.812
Lateral G.-Palmar G.	0.554	0.879	0.770
Lateral G.-Pronation	0.518	0.875	0.797
Palmar G.-Pronation	0.574	0.932	0.835
Palmar G.-Supination	0.552	0.915	0.830
Lateral G.-Supination	0.550	0.827	0.787
Pronation-Supination	0.502	0.807	0.771

**Table 9:** Classification Accuracy of Spectral Embedding for 2-Class Combinations.

	Spectral Embedding		
	SVM	k-NN	Naive Bayes
Hand O.-Lateral G.	0.588	0.868	0.715
Hand O.-Palmar G.	0.648	0.869	0.777
Hand O.-Pronation	0.603	0.869	0.810
Hand O.-Supination	0.629	0.875	0.751
Lateral G.-Palmar G.	0.657	0.881	0.744
Lateral G.-Pronation	0.587	0.892	0.768
Palmar G.-Pronation	0.545	0.845	0.710
Palmar G.-Supination	0.602	0.839	0.725
Lateral G.-Supination	0.635	0.908	0.790
Pronation-Supination	0.610	0.842	0.756

**Table 10:** Classification Accuracy of t-SNE for 2-Class Combinations.

	SVM	k-NN	Naive Bayes
Hand O.-Lateral G.	0.902	0.997	0.770
Hand O.-Palmar G.	0.900	0.995	0.769
Hand O.-Pronation	0.899	0.994	0.795
Hand O.-Supination	0.904	0.997	0.766
Lateral G.-Palmar G.	0.892	0.994	0.809
Lateral G.-Pronation	0.899	0.996	0.833
Palmar G.-Pronation	0.901	0.995	0.770
Palmar G.-Supination	0.939	0.996	0.726
Lateral G.-Supination	0.907	0.997	0.790
Pronation-Supination	0.870	0.996	0.792

**Table 11:** Classification Accuracy of MDS for 2-Class Combinations.

	SVM	k-NN	Naive Bayes
Hand O.-Lateral G.	0.795	0.905	0.794
Hand O.-Palmar G.	0.722	0.916	0.751
Hand O.-Pronation	0.836	0.901	0.795
Hand O.-Supination	0.706	0.936	0.744
Lateral G.-Palmar G.	0.773	0.912	0.805
Lateral G.-Pronation	0.800	0.918	0.847
Palmar G.-Pronation	0.738	0.881	0.761
Palmar G.-Supination	0.615	0.908	0.705
Lateral G.-Supination	0.743	0.962	0.770
Pronation-Supination	0.711	0.944	0.789

**Table 12:** Classification accuracy of LLE for 3-class combinations.

	SVM	k-NN	Naive Bayes
Hand O.-Pronation-Supination	0.415	0.802	0.663
Hand O.-Pronation-Palmar G.	0.417	0.802	0.702
Hand O.-Pronation-Lateral G.	0.351	0.813	0.699
Hand O.-Supination-Palmar G.	0.402	0.793	0.648
Hand O.-Supination-Lateral G.	0.381	0.820	0.664
Hand O.-Palmar G.-Lateral G.	0.378	0.877	0.635
Pronation-Supination-Palmar G.	0.397	0.738	0.608
Pronation-Supination-Lateral G.	0.361	0.825	0.620
Pronation-Palmar G.-Lateral G.	0.400	0.802	0.684
Supination-Palmar G.-Lateral G.	0.389	0.801	0.678

**Table 13:** Classification accuracy of Spectral Embedding for 3-class combinations.

	SVM	k-NN	Naive Bayes
Hand O.-Pronation-Supination	0.414	0.753	0.606
Hand O.-Pronation-Palmar G.	0.451	0.758	0.603
Hand O.-Pronation-Lateral G.	0.409	0.789	0.635
Hand O.-Supination-Palmar G.	0.429	0.726	0.612
Hand O.-Supination-Lateral G.	0.373	0.772	0.595
Hand O.-Palmar G.-Lateral G.	0.443	0.768	0.603
Pronation-Supination-Palmar G.	0.469	0.742	0.575
Pronation-Supination-Lateral G.	0.517	0.757	0.604
Pronation-Palmar G.-Lateral G.	0.500	0.777	0.600
Supination-Palmar G.-Lateral G.	0.465	0.707	0.578

**Table 14:** Classification accuracy of t-SNE for 3-class combinations.

	SVM	k-NN	Naive Bayes
Hand O.-Pronation-Supination	0.833	0.994	0.691
Hand O.-Pronation-Palmar G.	0.818	0.993	0.693
Hand O.-Pronation-Lateral G.	0.844	0.990	0.671
Hand O.-Supination-Palmar G.	0.803	0.993	0.637
Hand O.-Supination-Lateral G.	0.827	0.994	0.665
Hand O.-Palmar G.-Lateral G.	0.844	0.993	0.641
Pronation-Supination-Palmar G.	0.826	0.992	0.646
Pronation-Supination-Lateral G.	0.852	0.993	0.647
Pronation-Palmar G.-Lateral G.	0.856	0.992	0.650
Supination-Palmar G.-Lateral G.	0.846	0.992	0.652

**Table 15:** Classification accuracy of MDS for 3-class combinations.

	SVM	k-NN	Naive Bayes
Hand O.-Pronation-Supination	0.548	0.857	0.618
Hand O.-Pronation-Palmar G.	0.624	0.844	0.641
Hand O.-Pronation-Lateral G.	0.674	0.864	0.677
Hand O.-Supination-Palmar G.	0.540	0.868	0.568
Hand O.-Supination-Lateral G.	0.565	0.866	0.636
Hand O.-Palmar G.-Lateral G.	0.580	0.851	0.636
Pronation-Supination-Palmar G.	0.510	0.853	0.585
Pronation-Supination-Lateral G.	0.640	0.879	0.693
Pronation-Palmar G.-Lateral G.	0.640	0.851	0.679
Supination-Palmar G.-Lateral G.	0.499	0.862	0.652

**Table 16:** Classification accuracy for 5-class scenario.

Method	Classifier	Hand O.-Lateral G.-Palmar G.-Supination-Pronation
ISOMAP	SVM	0.900
	*k*-NN	0.933
	Naive Bayes	0.463
LLE	SVM	0.800
	*k*-NN	0.816
	Naive Bayes	0.463
Spectral E.	SVM	0.700
	*k*-NN	0.724
	Naive Bayes	0.453
t-SNE	SVM	0.800
	*k*-NN	0.809
	Naive Bayes	0.438
MDS	SVM	0.800
	*k*-NN	0.840
	Naive Bayes	0.467

**Table 17:** Detailed performance metrics for the best-performing model (t-SNE + *k*-NN).

Model	Accuracy (%)	AUC (95% CI)	Sensitivity	Specificity
t-SNE + *k*-NN	99.7	0.995 (0.992–0.998)	0.98	0.97

**Table 18:** Approximate computation time per trial for dimensionality reduction and classification (measured on a laptop with Intel i7 CPU, 16 GB RAM, MATLAB R2024a, and Orange 3.36).

Method	Dimensionality Reduction (ms)	Classification (ms)	Total Time (ms)
t-SNE + *k*-NN	∼320	∼30	∼350
ISOMAP + *k*-NN	∼80	∼10	∼90
MDS + *k*-NN	∼120	∼15	∼135
LLE + *k*-NN	∼150	∼15	∼165
Spectral Embedding + *k*-NN	∼100	∼10	∼110

## Data Availability

The data that support the findings of this study are available from the corresponding author upon reasonable request. Due to ongoing related studies, the raw data will be made publicly available after these studies are completed.

## Abbreviations

The following abbreviations are used in this manuscript:

EEG	Electroencephalography
BCI	Brain-Computer Interface
SCI	Spinal Cord Injury
MDS	Multi-Dimensional Scaling
ISOMAP	Isometric Mapping
LLE	Locally Linear Embedding
t-SNE	t-Distributed Stochastic Neighbor Embedding
SVM	Support Vector Machine
*k*-NN	k-Nearest Neighbors
CA	Classification Accuracy
AUC	Area Under the Curve
F1	F1-Score
Prec	Precision
PCA	Principal Component Analysis
RBF	Radial Basis Function
WPD	Wavelet Packet Decomposition
